# Post-Translational Modifications of Exosomal Proteins

**DOI:** 10.3389/fimmu.2014.00383

**Published:** 2014-08-11

**Authors:** Olga Moreno-Gonzalo, Carolina Villarroya-Beltri, Francisco Sánchez-Madrid

**Affiliations:** ^1^Vascular Biology and Inflammation Department, Centro Nacional de Investigaciones Cardiovasculares, Madrid, Spain; ^2^Servicio de Inmunología, Hospital de la Princesa, Instituto de Investigación Sanitaria de la Princesa, Universidad Autónoma de Madrid, Madrid, Spain

**Keywords:** post-translational modifications, exosomes, ubiquitination, sorting, multivesicular bodies

## Abstract

Exosomes mediate intercellular communication and participate in many cell processes such as cancer progression, immune activation or evasion, and the spread of infection. Exosomes are small vesicles secreted to the extracellular environment through the release of intraluminal vesicles contained in multivesicular bodies (MVBs) upon the fusion of these MVBs with the plasma membrane. The composition of exosomes is not random, suggesting that the incorporation of cargo into them is a regulated process. However, the mechanisms that control the sorting of protein cargo into exosomes are currently elusive. Here, we review the post-translational modifications detected in exosomal proteins, and discuss their possible role in their specific sorting into exosomes.

## Introduction

Post-translational modifications (PTMs) of proteins are biochemical changes generated after the synthesis of polypeptides on ribosomes. PTMs include changes to the chemical nature of aminoacid residues and also structural modifications that affect the interactive ability of proteins, and consequently their stability, subcellular localization, and activation state (1, 2). There are many types of PTM that can be classified according to the nature of the materials added: (1) a chemical group (phosphate, acetate, etc.), (2) carbohydrates, (3) lipids, (4) aminoacids, (5) other polypeptides, and (6) an isoprenyl group (Table 1). A protein can undergo many PTMs, changing its properties and broadening its capacity to adapt to cellular needs (2). Some modifications are reversible and are strictly regulated by the enzymes responsible for their addition or removal, acting as a dynamic switch that allows the cell to adjust protein functions according to requirements. Dysregulation of PTMs or mutation of modified residues are linked to disease, including cancer, neurodegenerative disorders such as Alzheimer, and cardiovascular disease, highlighting the importance of these protein modifications (3–7).

**Table 1:** **Post-translational modification of eukaryotic proteins**.

Groups of PTMs	Modification	Added group	Modified residues of target proteins	PTM in exosomal proteins	Biological relevance conferred by the PTMs	Reference
**POST-TRANSLATIONAL MODIFICATIONS OF EUKARYOTIC PROTEINS**						
Addition of a chemical group	Phosphorylation	Phosphate group	Tyr, Thr, Ser, His	FasL; AnnexinA2; tau; γ-synuclein	Sorting into exosomes; incorporation into exosomal membrane; spreading of toxic aggregates through exosomes; spreading of toxic aggregates through exosomes	(8–11)
	Acetylation	Acetyl group	Lys	–	–	–
	Methylation	Methyl group	Lys, Arg	–	–	–
	Oxidation	Different oxygen species	All amino acids, but preferentially Tyr, Phe, Trp, His, Met, Cys	γ-Synuclein	Spreading of toxic aggregates through exosomes	(11)
	Nitrosylation	Nitric oxide (NO)	Cys, Met	–	–	–
Addition of carbohydrates or glycosylation	N-linked glycosylation	Glycosyl group	Asn, Arg, and N-terminus	Several glycoproteins	Sorting of particular glycoproteins into exosomes (?)	(12–14)
	O-linked glycosylation	Glycosyl group	Ser, Thr, and amino acids in close proximity to Tyr phosphorylation sites	Several glycoproteins	Sorting of particular glycoproteins into exosomes (?)	(12–14)
	C-linked mannosylation and glypiation [glycosylphosphatidylinositol (GPI)anchor]	Glycosylpho sphatidylinositol (GPI) group	Carbon on a tryptophan side-chain and C-terminus, respectively	Several glycoproteins	Sorting of particular glycoproteins into exosomes (?)	(12, 13)
Addition of lipids (lipidation)	Palmitoylation	Palmitic acid	Cys	–	–	–
	N-myristoylation	Myristoyl group	N-terminal glycine residue	Artificial conjugation of TyA protein to myristoyl group	Sorting of TyA into shedding vesicles	(15)
Addition of amino acids	Polyglutamylation	Glutamic acid	Gly	–	–	–
Addition of other polypeptides	Ubiquitination	Ubiquitin protein	Lys, N-terminus, non-lysine residues (Cys, Thr, Ser)	Several proteins, some examples: LMP2A; PTEN; SIMPLE; HSP70; ARRDC1	Exosomal LMP2A loading; Sorting of PTEN into exosomes; Unknown function; Unknown function; Secretion into shedding vesicles	(16–20)
	SUMOylation (Small ubiquitin-related modifier addition)	SUMO1-4 proteins	Tetrapeptide consensus motif Ψ-K-x-D/E (Ψ: hydrophobic residue, K: lysine conjugated to SUMO, x: any amino acid, D or E: acidic residue)	hnRNPA2B1	Regulate the binding of miRNAs to hnRNPA2B1	(21)
	NEDDylation (neural-precursor-cell-expressed developmentally down-regulated 8 addition)	NEDD8 protein	Lys	–	–	–
	ISGylatyon (Interferon-stimulated gene 15 addition)	ISG15 protein	Lys	–	–	–
Isoprenylation	Farnesylation	Farnesyl group	Cys and sequence motifs CAAX, CC, or CAC at C-terminus (C: Cys, A: alanine, X: any amino acid)	–	–	–
	Geranylgeranylation	Geranylgeranyl isoprene unit	Cys and sequence motifs CAAX, CC or CAC at C-terminus (C: Cys, A: alanine, X: any amino acid)	–	–	–

A specific pattern of PTMs is detected in exosomes, 50–200 nm diameter vesicles secreted by most cells to the extracellular environment. Once released, exosomes can adhere to or be internalized by recipient cells, and in this way mediate cell-to-cell communication in a variety of contexts. Exosomes form through the invagination of the limiting membrane of specific endosomic compartments called multivesicular bodies (MVBs) (22). The resulting intraluminal vesicles (ILVs) are released as exosomes upon fusion of MVBs with the plasma membrane. Alternatively, MVBs can fuse with lysosomes, leading to degradation of their content. Exosomes have a specific composition of lipids, proteins, and RNAs; however, the mechanisms that control the sorting of molecules into these exosomal-proteins vesicles remain elusive. Here, we review the PTMs detected in exosomal proteins, and discuss their possible role in their specific sorting into exosomes.

## Ubiquitination and SUMOylation

Post-translational modifications increase the versatility of proteins by influencing their activation state, stability, subcellular localization, and ability to interact with other proteins. A particularly effective means of increasing protein versatility is the addition of ubiquitin, which can be attached to a target protein at a number of positions and in a variety of ways. The C-terminal glycine of ubiquitin usually forms an isopeptide bond with the ε-amino group of a lysine residue present in the target protein, resulting in mono-ubiquitination. In some cases, E4 ubiquitin ligases can add a poly-ubiquitin chain to a mono-ubiquitinated site (23). The equation becomes even more complicated considering that ubiquitin has seven lysines, and the fate of the target protein is determined by which lysine forms the link in the poly-ubiquitin chain: chains linked through lysine-48 (Ub-K48) label target proteins for degradation in the proteasome; Ub-K63 chains seem to be important for the DNA-damage response, endocytosis, autophagy, and signal transduction; Ub-K11 chains are implicated in endoplasmic-reticulum-associated degradation (ERAD); and Ub-K29 chains are involved in lysosomal degradation (24–33). Moreover, in some cases, ubiquitin can be linked through residues other than lysine, such as the N-terminal through the free amino group or the sulfhydryl group of cysteine residues (34).Ubiquitination can also compete with other PTMs, such as sumoylation or acetylation, and can enhance others such as phosphorylation (35–37).

**Figure 1:**
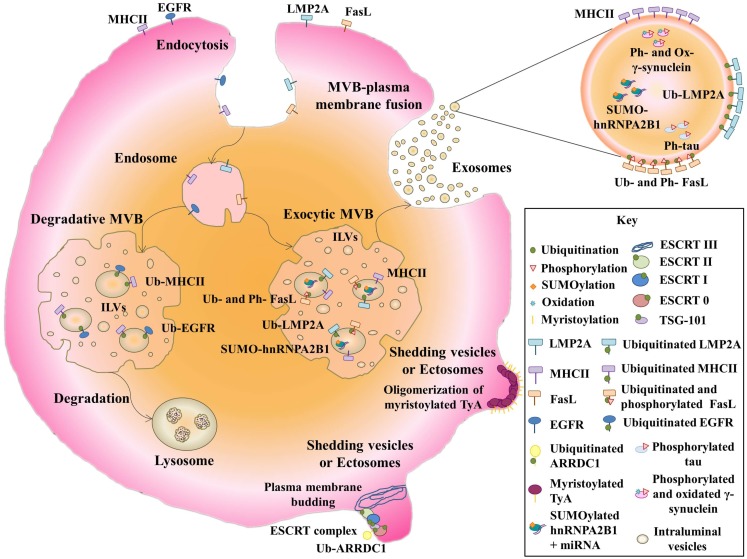
**Post-translational modifications of exosomal proteins**. Membrane receptors such as EGFR and MHCII are ubiquitinated and sorted to MVBs. Then, they follow a degradative pathway by the fusion with lysosomes. Note that non-ubiquitinated-MHCII can be sorted into exosomes. Ubiquitinated LMP2A and ubiquitinated and phosphorylated FasL follow a secretory pathway where both modified proteins are delivered into exosomes. Non-membrane proteins like SUMOylated hnRNPA2B1, phosphorylated and oxidized γ-synuclein, and phosphorylated tau are packed into exosomes. Myristoylated TyA protein is able to oligomerize, leading to the formation of shedding vesicles. Ubiquitinated ARRDC1 can induce plasma membrane budding by an ESCRT complex-depending mechanism, producing ectosomes.

Ubiquination, thus denotes a complex network of PTMs, and its role in the sorting of proteins into exosomes is far from understood. There seems to be consensus that ubiquitination is necessary for sorting proteins into ILVs destined for degradation through the fusion of the encompassing MVB with lysosomes. This process is mediated by the endosomal sorting complex required for transport machinery (ESCRT complex) and affects proteins such as epithelial growth factor receptor (EGFR) (38) (Figure 1). This machinery recognizes ubiquitinated cargoes and catalyzes the abscission of endosomal invaginations, forming ILVs that contain the sorted cargo [reviewed in Raiborg and Stenmark (39)]. The ESCRT complex consists of four subcomplexes, ESCRT-0, -I, -II, and -III, and several accessory proteins. ESCRT-0, -I, and -II contain ubiquitin-binding subunits that interact directly with ubiquitinated cargo. The directional flow of cargo from ESCRT-0 to ESCRT-I and -II might be regulated by PTMs. In fact, the ESCRT-0 subunits are known to be phosphorylated and to be mono-ubiquitinated (40–42). The latter modification keeps these subunits in an inactive form owing to intramolecular interactions between their ubiquitin interacting motifs and the appended ubiquitin (43, 44). However, the role of ubiquitin and the ESCRT complex in the sorting of proteins into ILVs for exosome secretion is still unclear, and MVB biogenesis, exosome secretion, and exosomal-protein sorting have been reported in an ESCRT/ubiquitin-independent manner [reviewed at Villarroya-Beltri et al. (45)].

The Epstein-Barr virus (EBV) protein LMP2A (latent membrane protein 2A) is ubiquitinated in exosomal fractions (16). LMP2A contains two PXYY motifs, through which it associates with neural precursor cell expressed developmentally down-regulated protein 4 (Nedd4)-family ubiquitin ligases (16). Ubiquitination of LMP2A leads to endocytic transport of the protein from the plasma membrane to MVBs. Nedd4 E3 ubiquitin ligases are able to bind directly to target proteins though the PPXY motif, but proteins lacking this motif can bind Nedd4 through the adaptor Nedd4-family-interacting protein 1(Ndfip1), leading to their ubiquitination (46). Ndfip1 is involved not only in protein degradation, but also in protein traffic to exosomes (46). Ndfip1 overexpression increases the protein content of exosomes and enhances exosomal sorting of normally absent proteins, such as Nedd4, Nedd4-2, and Itch. These exosomal proteins moreover appear to be highly ubiquitinated, suggesting that Ndfip1 transfers other ubiquitinated proteins to exosomes (46) (Figure 1). For example, the Ndfip1 adaptor function is required for exosomal export of phosphatase and tensin homolog deleted on chromosome 10 protein (PTEN) ubiquitinated on lysine 13 (17).

With other proteins, however, ubiquitination appears to be unimportant or inhibitory to exosomal export. Secretion into exosomes of small integral membrane protein of the lysosome/late endosome (SIMPLE) is enhanced by mutations in its PPXY motif, which mediates its binding to E3 ubiquitin ligases (18). Ubiquitination is also not required for the packaging of major histocompatibility complex II (MHC-II) into exosomes (47). The use of a chimeric-ubiquitinated-MHC-II molecule does not specifically lead these molecules into exosomes, and forcing MHC-II ubiquitination by expression of membrane-associated ring finger (C3HC4) 8 (MARCH) E3 ubiquitin ligase does not enrich MHC-II molecules in exosomes, though it does completely deplete them from the plasma membrane. Moreover, directed-mutagenesis of all MHC-II lysine residues does not impair the exosome sorting of these receptors (47). However, ubiquitination of the MHC-II cytoplasmic domain, required for recognition by the ESCRT complex, is important for sorting membrane MHC-II to MVBs for lysosomal degradation (48). The non-ubiquitination of MHC-II molecules present in exosomes suggests that this PTM is not involved in sorting to these vesicles. The two mechanisms for loading MHC-II into MVBs are engaged for different physiological functions. Thus, whereas ubiquitin-dependent sorting takes place in immature DCs, in which ubiquitinated receptors are degraded in lysosomes, in activated-DCs, non-ubiquitinated MHC-II-containing exosomes are efficiently delivered to interact with T cells, enhancing antigen specific MHC-II-mediated presentation (48) (Figure 1). Heat shock protein 70 (HSP70) also seems to be sorted into exosomes independently of its ubiquitination. Thus, although deletion of the deubiquitin domain of COP9 signalosome complex subunit 5 (CSN5) enhances packing of ubiquitinated HSP70 into exosomes, knockdown of the entire CSN5 protein increases the levels of both modified and non-modified HSP70 in exosomes (19).

Mass spectrometry analysis of PTMs in extracellular vesicles released by insulinoma cells identified multiple poly-ubiquitinated proteins (49). Enrichment of exosomes in poly-ubiquitinatied proteins was also demonstrated by an approach based on the use of FK1 antibody (which only binds poly-ubiquitinated proteins) and P4D1 (which labels poly- and mono-ubiquitinated proteins) (50). Other studies suggest that exosomal proteins are preferentially mono-ubiquitinated or de-ubiquitinated, based on western analysis showing discrete ubiquitinated protein bands rather than smeared bands (19).

Ubiquitination has been shown to be important for the secretion of a novel type of extracellular vesicle, distinct from exosomes, called arrestin-domain-containing protein 1(ARRDC1)-mediated microvesicles (ARMMs). ARMMs directly bud from the plasma membrane upon interaction of the tumor susceptibility gene 101 protein (TSG101) with a PSAP motif in ARRDC1, which is localized through its arrestin domain at the plasma membrane (20). ARRDC1 in vesicles is ubiquitinated by the E3 ligase WW domain-containing protein 2 (WWP2). Down-regulation of WWP2 decreases ARRDC1 protein level in vesicles, and a PPXY-mutant of ARRDC1 strongly inhibits ARMM secretion, suggesting that ARRDC1 ubiquitination promotes ARRDC1 sorting into vesicles and ARMM secretion (20) (Figure 1).

Another ubiquitin-like modifier called small ubiquitin-related modifier (SUMO) has been found to modify the exosomal-protein heterogeneous nuclear ribonucleoprotein A2B1 (hnRNPA2B1). This modification affects the ability of this protein to export micro ribonucleic acids (miRNAs) into exosomes, probably by affecting its binding to miRNAs (21). hnRNPA1 in exosomes was also found to be modified, increasing its molecular weight by about 12 kDa on gel electrophoresis (21) (Figure 1). This change in molecular weight of hnRNPA2B1 and other proteins has been shown before (49).

## Other PTMs: Phosphorylation and Glycosylation

Mass spectrometry analysis of extracellular vesicles also detects phosphorylated proteins (49). Phosphorylation and ubiquitination co-regulate sorting of Fas ligand (FasL) into secretory lysosomes by controlling its entry into MVBs (8). FasL contains a proline-rich domain (PRD) in the cytosolic tail to which tyrosine kinases, such as FGR, FYN, and LYN bind, and phosphorylation of tyrosine residues by these kinases enhances internalization to MVBs. The flanking regions of the PRD contain lysines, which are mono-ubiquitinated. Mutation of these lysines impairs the localization of FasL in MVBs, but mutation of the tyrosines does not affect mono-ubiquitination. Phosphorylation is thus not required for ubiquitination, but both PTMs are necessary for incorporation of FasL into to MVBs (8) (Figure 1).

Phosphorylation is also involved in incorporation of the Ca2^+^-dependent phospholipid-binding protein Annexin A2 into exosomal membranes, through the action of raft-resident kinases, such as SRC or LYN on Tyr-23 (9). Aberrant phosphorylation of the protein tau on threonine-181 promotes its incorporation into exosomes, resulting in the spreading of this abnormally processed protein in Alzheimer disease patients (10).

The protein γ-synuclein is transported in exosomes in its modified form. This modification consists of oxidation of Met-38 and Tyr-39, which confers prion-like properties and causes the formation of toxic aggregates. The spreading of these aggregates is in part mediated by the exosomal transport of oxidated-γ-synuclein to glial cells (11) (Figure 1).

Carbohydrate modifications, involved in protein trafficking, cellular recognition, and communication of cells with their extracellular environment, have also been studied in extracellular vesicles (12, 13, 51). Vesicles of diverse cells types are enriched in proteins with high mannose, polylactosamine, α-2,6-sialic acid, and complex N-linked glycans adjuncts; in contrast, there is a comparative under-representation of specific glycan epitopes, such as terminal blood group A and B antigens (12, 13). Exosome glycan profiles of different cell sources, such as T-cells, melanoma and colon cancer cells, and biological fluids like breast milk, are very similar, although they conserve some features of their parent membranes (13). The carbohydrate fingerprint detected in exosomes is less diverse than that observed in parent cells, but correspond to a conserved fraction of the parent cellular membrane that display a particular glycan profile (13). The variability observed between cellular and exosomal carbohydrate signatures has been suggested to indicate different membrane microdomain origins of these vesicles (13). It has been described that polyLacNac and high mannose modifications associated with galectins and VIP36 are responsible for the oligomerization of glycoproteins that mediate their sorting into Golgi-derived vesicles (12, 52–55). Galectins and galectin-associated proteins have also been detected in exosomes so it is possible that glycosylation may also play a role in the sorting cargo into extracellular vesicles (12, 13).

Membrane anchors have also been shown to be important for the budding of vesicles derived from the plasma membrane. An N-terminal acylation tag serves as a signal for the import of highly oligomeric cytoplasmic proteins, like the yeast protein TyA, into shedding vesicles (56). The membrane anchor that most effectively promotes TyA budding is myristoylation. However, targeting of TyA to the endosomal membrane by fusion to a Phosphatidylinositol-3-phosphate (PI3P) binding domain does not produce the same effect (15) (Figure 1).

## Concluding Remarks

Post-translational modifications decorate proteins and drive their fate in cells by affecting multiple parameters including stability and localization. Different modifications can affect the same protein; sometimes competing with each other or being mutually exclusive, but in other cases can promote other modifications. Enzymes controlling PTMs additionally show very specific patterns of expression, activation, and subcellular localization, exponentially increasing the diversity and potentiality of cellular proteomes.

Different types of PTMs have been found in exosomal proteins; however, the role of these modifications in the localization of proteins into exosomes is not clear. The enigmatic role of ubiquitination, whose final consequences seem to differ depending on the target protein, is a particular case in point. The type of ubiquitination may also account for the fate of the modified protein, and could be a key determinant for its loading in exosomes. In some cases, ubiquitination seems to target the protein into MVBs destined for degradation, whereas sorting of proteins into MVBs that fuse with the plasma membrane to release exosomes seems to be ubiquitin-independent, clearly pointing to the existence of different types of MVB with different sorting mechanisms.

Specific protein modifications can reflect a particular pathological condition. The presence of modified proteins in exosomes can therefore make them invaluable tools for diagnosis, since modifications could be easily detected in exosomes obtained from body fluids without the need for invasive tissue biopsies.

## Conflict of Interest Statement

The authors declare that the research was conducted in the absence of any commercial or financial relationships that could be construed as a potential conflict of interest.
